# The Case of a Man, Who Died in Consequence of the Bite of a Rattle-Snake; with an Account of the Effects Produced by the Poison

**Published:** 1810-11

**Authors:** Everhard Home


*The Case of a Man, who died in consequence of the Bite of a Rattle-Snake; with an Account of the Effects produced by the Poison.—By* EVERHARD HOME, Esq. F. R. S.—*with preliminary Observations.*

THE public sensation which was occasioned by a man having been bitten by a *rattle-snake,* kept at a menagerie in Piccadilly, was extended as far as a knowledge of the fact had spread. Curiosity, with respect to the symptoms and fate of the wounded man, was highly excited; but many months elapsed before this curiosity was satisfied. A minute detail of the case has now been published, and we embrace this opportunity of laying it before our readers.

The *genus* to which the serpent that wounded the subject of the following case, belongs, is peculiar to America, and has the expressive appellation CROTALUS, given to it by Linnæus. It contains five species, according to the *Systema Naturæ.*—LINN.

1 CROTALUS *miliarius.*
2 ————— *horridus.*
3 ————— *dryinas.*
4 ————— *durissus.*
5 ————— *mutus.*

To which the *Count la Capede* has added a sixth, the Fisher, *(le Piscivoire)* described by Catesby, *(Nat. Hist. of Carolina,)* and by him only, and two varieties. Gronian durissus. *(Crotalophorus tertius.—Gron. Mus.* 11. 70. *n.* 45.*)* and the fasciated durissus. *(Crotalus faciatus. Vosmaer. monagr. Anno* 1767.*)* The whole of the species of this *genus* are furnished with poisonous fangs, but have the character of seldom biting,* unless irritated, or for the purpose of securing their prey.

The species by which this person was bitten, is the *Crotalus horridus*, the most terrific and formidable of the genus.—Many names have been given to it, either expressive of its mechanism, or its qualities; it is the *Caudisona terrifica* of Laurentius; the *Vipera caudisona*, and *Anguis crotalophorus* of our countryman Ray; the *Regina serpentum* of Hernandez; the *Boiquira* of Piso, and the Encyclop. Method:---From the quickness of its motions, the Mexicans call it *Ecacoatle*,† for it

* The pacific disposition of the rattle-snake is asserted by Batram.

† This word, expressive of velocity, is sometimes written *Hoatcoatl.* Savage nations often employ a highly figurative language. The Mexicans denominate the rattle-snake *Teutlacacauqui,* i. e. the king of snakes.

darts darts on its prey, wounds, and retires, with the swiftness of the wind.

The *Crotalus horridus* is found along the American continent, from the Streights of Magellhan, to Lake Champlain, in Canada. For a long time after the discovery of the New World, this *Regina serpentum*, reigned without controul over its vast wilds and impassable forests. Few, possibly none, of the native animals preyed upon it; and it was secured from the destroying hand of man, by superstitious terrors, that influenced, unaccountably indeed, the ancient Americans. As human dominion extends, the lower orders of nature submit, or are exterminated. Thus the arts and labour of civilized life, as they clear and cultivate the soil of America, daily diminish the numbers of the *Crotalus;* and contract the space in which its ravages are exercised. It is probable the time is not far distant when it may become a rare animal even in America.*

The habits and powers of the rattle-snake, (*Crotalus horridus*, for it is this species of the genus, upon which observation has been most exercised) have been examined with anxious precision; and much has been written to elucidate them; but on some of its striking properties doubt still remains. There are four particulars, which have most employed attention :---its poison---its power of fascination---its rattle---and its abominable fœtor.

The general terror excited by the rattle-snake, is described with wonderful force and picturesque effect, by the elegant Buffon. "A traveller," says he, "wandering in the burning desarts of Africa, who hears the roaring of a famished tyger furious for prey, and who has no means either of escape or defence, feels not greater dread than when passing through the vast forests of the warm and humid regions of the New World, he scents the abominable fœtor of the *Boiquira*, hears the terrifying noise of its portentous rattle, and observes it ready to spring upon him, from amid the verdant foliage and brilliant flowers of these delightful solitudes." Again, he says, "During storms of thunder it is most to be dreaded. One even shudders in idea, at the terrible situation and mortal agony of a person who, escaping in the midst of almost total darkness from the terrors of a tornado, seeks shelter under the ledge of a rock, and perceives the glaring eyes of a *Boiquira* through the gloom, sometimes illuminated by the lightning, while he hears the rattle of its tail, and the horrid hissings of its venomous mouth. Language has not power to express the deadly swiftness with which the poison of the

* It is a remarkable circumstance, that the European Hog should be the most formidable instrument for the destruction of this serpent. The moment a hog scents a rattle-snake, he erects his bristles, rushes upon it with avidity, and seizes it with extraordinary address.

*Boiquira*

*Boiquira* sometimes acts. In a minute, half a minute, in an instant, the bitten animal has expired.* In this country, a rattle-snake bit dogs that died in a few minutes. These experiments were made at the College of Physicians, about 1728. Mr. Ranby, the Surgeon, examined the poisonous apparatus, and gave a description of it in the Phil. Trans. Vol. 35. p. 377. Dr. Edward Tyson had, some years before this, made a minute dissection of another that had been sent to this country, about 1690; and which, probably, was the first brought hither alive. (Phil. Trans. 13. 25.) Of the chemical qualities of the venom not much is known. It is described, however, as a greenish fluid, and that it tinges linen of a green colour, which becomes brighter by washing. It is believed that the poison sometimes accumulates in its receptacle, to a degree that becomes painful to the animal; and that then it attacks every thing that comes in its way.---*(Hist. Gen. dà Voyage).*

Of the facinating power of the *Crotalus horridus*, much has been related, but accompanied with circumstances so extravagant, as to gain but little credence with the reflecting part of mankind. An ingenious and learned naturalist of America (Dr. Barton) has written a treatise expressly on the subject; in which he adduces many observations to prove the non-existence of this power.— The fascinating property has been supposed by some to be connected with the stupifying quality in the breath of the *Boiquira;* by others, to be effected by the dazzling splendour of its eye. *Kalm* offers a solution of some of the instances. He conjectures that " in most of those instances, where a squirrel or a bird has been seen to come apparently of its own accord from the top of a tree into the mouth of the *rattle-snake*, that it had been previously bitten by the serpent; and having escaped into the tree, where it expressed, by its cries and agitation, the violent action of the venom; that as its strength diminished, it gradually descended from branch to branch, till at length it became exhausted, and fell to the ground near the serpent, which remained steadfastly following all its motions with its eyes, and ready to seize it whenever it came within reach."

The mechanism of the rattle has been minutely described; and its structure is doubtless a subject worthy of investigation; but why the animal should have an appendage that must necessarily give notice of its hostile intention, is a question not easily solved. It has been understood, that this serpent could not move without its rattle giving notice of its being in action. This did not, however, appear to be precisely the fact in the two rattle-snakes at Frazer's nursery, Chelsea, in the summer of 1809,

* In the case related by Mr. Home, it may be observed, though the patient lived some weeks, the sensorium was immediately effected.

and and by one of which, the man whose case is here related was bitten. From observing the habits of those, we can say that when irritated, they formed themselves into several coils, the head erect in the centre, and the tail, with the rattle at the circumference. In this position the rattle was moved with a quick and tremulous motion, to be heard at thirty yards distance.--- This motion of the rattle, which is effected with extreme velocity, appears to mark the design to strike when the object comes within the proper distance; and certainly manifests the snake then to be in a hostile position. If in the common movements this apparatus gives any audible sound, it will have a different character from that which indicates anger. The stroke of the snake, and the cessation of the action of the rattle, occurred at the same instant.

The fœtid odour that is emitted from this serpent, if it sometimes deprives small animals of the power to escape: at others serves as a warning of its approach; and horses, cattle, and other animals thus escape from its bite. This disgusting scent has been described as a property in the breath, and exhaled from the lungs; it is probable, however, that it is afforded by some glands described by Tyson, and placed near the anus: or is compounded of the fœtor arising from the fluid secreted by these glands, and the kalitus thrown out by the lungs and general surface.

Many remedies for the bite of the rattle-snake have been recommended, particularly the *Polygala Senega*; and several instances are recorded of their success. In the case before us, those employed were given on general indications, rather than with a view to specific properties.

When we request the attention of our readers and correspondents to a subject of such interest and importance as the *animal poisons*, and especially those of the order *Serpentes;* we expect that the natural history and deleterious effects of those poisons, will be elucidated in a proportion agreeing with the investigation we hope to elicit.

*The Case of a Man who was bitten by a rattle-snake, &c. &c.* *(Phil. Trans. for* 1810.—*Part* 1.*)*

Thomas Soper, 26 years of age, of a spare habit, on the 17th of Oct. 1809, went into the room in which two healthy rattle-snakes, brought from America, in the preceding summer, were exhibited. He teized one of them, with the end of a foot rule, but could not induce the snake to bite it, and on the rule dropping out of his hand, he opened the door of the cage to take it out; the snake immediately darted at the hand, and bit it twice in succession, making two wounds on the back part of the first first phalanx of the thumb, and two on the side of the second joint of the fore finger. The snake is between four and five feet long, and when much irritated bites the object twice, which I believe snakes do not usually do.

The bite took place at half past two o'clock. He went immediately to Mr. Hanbury, a chemist in the neighbourhood.—There was at that time no swelling on the hand, and the man was so incoherent in his language and behaviour, that Mr. Hanbury considered him to be in a state of intoxication, and gave him a dose of jalap to take off the effects of the liquor, and made some slight application to the bites. It appeared, on enquiry, that the man had been drinking, but that before he was bitten, there was nothing unusual in his behaviour. After leaving Mr. Hanbury the hand began to swell, which alarmed him, and he went to St. George's Hospital. He arrived there at three o'clock. The wristband of his shirt had been unloosed, and the swelling had extended half way up the fore-arm before his admission. The skin on the back of his hand was very tense, and the part very painful. At four o'clock the swelling extended to the elbow, and at half past four it had reached half way up the arm, and the pain had extended to the axilla. At this time, Mr. Brodie, who visited him in my absence, first saw him; he found the skin cold; the man's answers were incoherent: his pulse beat 100 strokes in a minute, and he complained of sickness. Forty drops of aqua ammoniæ puræ, and thirty drops of spiritus ætheris vitriolici in an ounce of mistura camphorata, were given to him, but did not remain on his stomach. The wounds were bathed with the aqua ammoniæ puræ, and the arm and fore-arm had compresses wetted with camphorated spirits applied to them. At five o'clock he took two drams of spiritus ammoniæ compositus, and thirty drops of æther in an ounce and a half of mistura camphorata, which remained on his stomach. At six o'clock his pulse was stronger; at half past seven his pulse was very feeble, and thirty drops of æther, and the same quantity of aqua ammoniæ puræ were given in water. At half past eight it was repeated. At nine o'clock he had the feeling of great depression, his skin was cold, his pulse weak, beating 80 strokes in a minute. The dose was increased to fifty drops of both medicines, and repeated. At a quarter past ten o'clock the pain had become very violent in the arm; his pulse was stronger, but fits of faintness attack him every fifteen minutes, in which the pulse was not perceptible, but in the interval his spirits were less depressed. In the course of the evening he had two stools. At half past eleven o'clock I first saw him. The hand, wrist, fore-arm, and arm, were much swelled up to the top of the shoulder, and into the axilla. The arm was quite cold, and no pulse could be felt in any part, not even in the axilla, the swelling preventing me from feeling the axillary axillary artery with any degree of accuracy. The wounds made on the thumb were just perceptible; those on the finger were very distinct. His skin generally was unusually cold. I took some pains to diminish his alarm of danger, and found his mind perfectly collected: he said he hoped he should recover. At one o'clock in the morning of the 18th, he talked indistinctly: his pulse beat 100 in a minute; the attacks of faintness came on occasionally. The medicine was repeated every hour. At eight o'clock in the morning of the 18th, his pulse beat 132 strokes in a minute, and was very feeble. The swelling had not extended beyond the shoulder to the neck, but there was fullness down the side, and blood was extravasated under the skin as low as the loins, giving the back on the right side a mottled appearance. The whole arm and hand was cold, but painful when pressed; the skin was very tense; on the inside of the arm below the axilla, and near the elbow, vesications had formed; and under each of the vesications there was a red spot in the cutis, of the size of a crown piece. The skin generally over the body had become warm. He was low and depressed; there was a tremulous motion of his lips, and the faintings recurred at nearly the same intervals as in the preceding evening. The last dose of medicine was rejected by vomitting, but some warm wine remained on his stomach. The arm was fomented. At twelve o'clock, in addition to the above symptoms, there was a starting of his limbs. He had attempted to take some broth, but his stomach did not retain it. The skin of the whole arm had a livid appearance, similar to what is met with in a dead body, when putrefaction has begun to take place, unlike any thing which I had ever seen in so large a portion of the living body. An obscure fluctuation was felt under the skin of the outside of the wrist, and fore-arm, which induced me to make a puncture with a lancet, but only a small portion of a serous fluid was discharged. My colleague, Dr. Nevinson, was present at this visit, and we agreed to continue the internal use of the volatile alkali, with the view of rousing the stomach to action, not considering it as having any specific power over the poison. At eleven o'clock in the evening, finding that his stomach did not always retain the medicines, nor even small quantities of brandy, which were given him, I directed the volatile alkali to be left off, and two grains of opium to be given, and repeated every four hours. At this time his pulse was scarcely perceptible at the wrist, the fainting fits were not less frequent. The vesications and red spots were increased in size.

October 19. At nine o'clock in the morning his pulse was scarcely perceptible: his extremities were cold; the vesications were larger, and size of the arm was diminished. He was drowsy, probably from the effect of the opium. He had taken nothing but brandy during the night. At three o'clock in the afternoon he he was more depressed: spoke only in whispers: the vesications were increased: the fainting fits less frequent. The arm was diminished in size, and he had sensation in it down to the fingers. At eleven o'clock at night his pulse beat 130 in a minute, and was low. The opium was left off. A stool was procured by clyster. He was ordered to have a glass of camphorated mixture occasionally, and wine and brandy, as often as he could be induced to take them.

October 20. He had dozed at intervals during the night; his spirits were better, and his extremities warmer. At nine o'clock he took coffee for breakfast. He afterwards took some fish for dinner, but it did not remain on his stomach; he therefore took brandy and coffee at intervals, half an ounce at a time, as larger quantities did not remain on his stomach.

October 21. He had slept at intervals during the night, but was occasionally delirious: his pulse 120 in a minute. Brandy and jelly were the only things that stayed on his stomach. The size of the arm was reduced, but the skin was extremely tender.

October 22. He had slept during the greater part of the night, his pulse beat 98 in a minute; he took some veal for dinner, and brandy at intervals. In the evening his pulse became full and strong: he was ordered wine instead of brandy. The right side of the back down to the loins was inflamed and painful; and had a very mottled appearance, from the extravasated blood under the skin.

October 23. His pulse continued full, and the arm was very painful, though reduced in size. The vesications had burst, and the exposed cutis was dressed with white ointment. Stools were procured by an opening medicine. He took some veal and porter for dinner; the wine was left off. In the evening he had a saline draught with antimonial wine.

October 24. There was no material change.

October 25. His pulse had increased in frequency, but in other respects he was nearly the same. His bowels were opened by medicine.

October 26. The arm was more swelled and inflamed.

October 27. The inflamation of the arm had increased: his tongue was furred, and his pulse was very frequent. He attempted to sit up, but the weight of the arm and the pain prevented him. The arm was bathed with spirits of wine and aqua ammoniæ acetatæ in equal quantities.

October 28. A slough had begun to separate from the inside of the arm below the axilla, and a purging had come on, for which he was ordered chalk mixture and laudanum. In the night he had a rigor.

October 29. The purging had abated: his pulse beat 100 in a minute, and was feeble. A large abscess had formed on the outside of the elbow, which was opened, and half a pint of reddish-brown matter was discharged with sloughs of cellular membrane floating in it. The lower part of the arm became much smaller, but the upper part continued tense. A poultice was applied to the wound. The lower portion of the arm and the fore-arm were covered with circular stripes of soap cerate. He was ordered to take the bark, and allowed wine and porter.

October 30. The redness and swelling of the upper part of the arm had subsided: the pulse was 100 in a minute. The purging had returned. The bark was left off: the chalk mixture and laudanum were given, and an opiate clyster administered.

October 31. The pulse beat 120 in a minute. The discharge from the abscess had diminished, the purging continued, and at night he had a rigor.

November 1. The pulse was 120. His voice was feeble; he had no appetite, was delirious at intervals. Ulceration had taken place on the opening of the abscess, so that it was much increased in size. He drank two pints of porter in the course of the day.

November 2. His pulse was very weak; his countenance was depressed; his tongue brown; the ulceration had spread to the extent of two or three inches. Mortification had taken place in the skin nearer the axilla. His stomach rejected every thing but porter; in the night he was delirious.

November 3. The mortification had spread considerably: the purging continued: the fore-finger which had mortified, was removed at the second joint.

November 4. He died at half-past four o'clock in the afternoon.

Sixteen hours after death, the body was examined by Mr. Brodie and myself, in the presence of Mr. Maynard, the house surgeon, and several of the pupils of the hospital.

With the exception of the right arm which had been bitten, the body had the natural appearance. The skin was clear and white, and the muscles contracted.

The wounds made by the fangs at the base of the thumb were healed, but the puncture made by the lancet at the back of the wrist was still open. That part of the back of the hand, which immediately surrounded the wounds made by the fangs, for the extent of one inch and a half in every direction, as also the whole of the palm, was in a natural state, except that there was a small quantity of extravasated blood in the cellular membrane. The orifice of the abscess was enlarged, so as to form a sore on the outside of the arm, elbow, and fore-arm, near six inches in length. Around this, the skin was in a state of mortification, more than half way up the outside of the arm, and as far downwards, wards, on the outside of the fore-arm. The skin still adhered to the biceps flexor muscle in the arm, and flexor muscles in the fore-arm, by a dark coloured cellular membrane. Every where else in the arm and fore-arm, from the axilla downwards, the skin was separated from the muscles, and between these parts there was a dark coloured fluid, with an offensive smell, and sloughs of cellular membrane resembling wet tow, floating in it. The muscles had their natural appearance every where, except on the surface, which was next the abscess. Beyond the limits of the abscess, blood was extravasated in the cellular membrane, and this appearance was observable on the right side of the back as far as the loins, and on the right side of the chest over the serratus major arnicus muscle.

In the thorax the lungs had their natural appearance. The exterior part of the loose fold of the pericardium where it is exposed, on elevating the sternum, was dry, resembling a dried bladder. The cavity of the pericardium contained half an ounce of serous fluid, which had a frothy appearance from an admixture of bubbles of air. On cutting into the aorta, a small quantity of blood escaped, which had a similar appearance. The cavities of the heart contained coagulated blood.

In the abdomen, the cardiac portion of the stomach was moderately distended with fluid: the pyloric portion was much contracted; the internal membrane had its vessels very turgid with blood. The intestines and liver had a healthy appearance. The gall-bladder was moderately full of healthy bile. The lacteals and thoracic duct were empty; they had a natural appearance.

In the cranium, the vessels of the pia mater and brain were turgid with blood; the ventricles contained rather more water than is usual, and water was effused into the cells, connecting the pia mater and tunica arachnoides. It is to be observed, that these appearances in the brain and its membrane are very frequently found in cases of acute diseases which terminate fatally.

The following cases were sent from India, to my late friend, Dr. Patrick Russell; they arrived after his death, and Mr. Claude Russell very kindly gave them to me, knowing the subject of them to be one, in which I had taken an interest. As they correspond in many of the circumstances, with that which has been detailed, I have inserted them in this place, as well as an experiment, which I had an opportunity of making in the West Indies, on the effects of the snake's poison on animals.

A boy, a slave of a gentleman in India, was bitten by a snake called Kamnlee by the natives, in the lower part of the arm, at eight o'clock in the evening. The blood flowed very freely for some time. He died next day at noon in great pain.

A sepoy, sixty years of age, was admitted into the hospital of his regiment, under the care of Mr. Perrin, assistant surgeon, at

 four four o'clock in the afternoon of the 15th of October, 1802, in consequence of his being bitten by a Cobra di capello, on the back part of the hand. At the time of his admission he complained of pain running up the arm. He immediately took a drachm of eau de luce, and this dose was repeated every half hour, and the same remedy was applied externally as a lotion to the arm and fore-arm. At four o'clock in the morning of the 16th of October, the pain began to increase, and the arm to swell with great hardness and stiffness, and tumor in the axilla, with much inclination to vomit. He took twelve grains of Dr. James's powder, which brought up a great quantity of bilious matter. He drank copiously of warm water, but no perspiration was induced. He appeared relieved for a short time. At eight o'clock in the morning the arm was distended, painful, and discoloured. He took four ounces of brandy, and repeated it every hour until twelve o'clock, with a drachm of eau de luce occasionally. At this time he was a little revived. The brandy was reduced to two ounces, which were carefully and regularly given every hour, until twelve at noon on the 17th of October, when the arm was more free from pain, but much swelled, hard, and black: his spirits and pulse also were considerably relieved. The eau de luce was now omitted, but the brandy was continued every hour, until twelve o'clock at noon on the 18th of October, when the stiffness and tumor in the axilla had disappeared, the arm was still swelled, but was softer, and less painful. The brandy was omitted; at night he took six grains of Dr. James's powder. On the 19th of October the arm was less, softer, with little or no pain; a blister was formed and burst on the back of the hand, which discharged three ounces of black fœtid pus. On the 20th, an abscess burst on the hand, in the same situation as the blister, which discharged a large quantity of fluid, having an offensive smell. He was directed to take a drachm of Peruvian bark in port-wine, every two hours. On the 22d the swelling was gone, but the discharge was considerable. From this time the man gradually, but slowly recovered, with the loss of the use of his fore-finger, which remained permanently extended, and some of the other fingers were affected in a less degree.

In this case, the swelling of the arm was slower in coming on, and less extensive; the pain running up to the axilla, which preceded it, was mistaken for the effect of absorption.

In the year 1782, while in the island of St. Lucia, I made the following experiment.

A spotted dark-coloured snake, about two feet in length, having the poison fangs on each side double, with the corresponding surfaces grooved, so as to form a canal for the poison, was put into a square tin box, open at the top, in which a half-grown rat was confined. The rat expressed great terror, and remained crouching crouching in one corner of the box, with its eyes fixed on the snake, who lay coiled up at some distance; they were allowed to remain a few minutes in this situation. I then raised one end of the box, which caused the snake to slide along the smooth surface, till it came in contact with the rat, which it immediately bit. The rat died in a minute after the bite. I removed it immediately from the box by means of a long pair of forceps. The wounds made by the fangs were marked by two specks of blood immediately below the shoulder blade. On dividing the skin with a scalpel, the cellular membrane under it was found entirely destroyed: the muscles were detached from the ribs, and from a small portion of the scapula. The parts immediately surrounding the bite were exceedingly inflamed; as far as I could trust to memory, the appearances very much resembled those produced on the muscles of a dog's thigh, by the application of white arsenic, in consequence of which, death ensued in about sixteen hours.

Fifteen hours after the death of the first, a second rat was bitten by the same snake. This rat was much irritated, and bit the snake in the neck, so violently, that the latter died in about ten minutes. The rat continued very lively for about six hours, and then died. On examination after death, the bite was found to have been inflicted on the left side of the navel, and the abdominal muscles at that part were in the same state as in the other rat, but in a less degree.

It appears from the facts, which have been stated, that the effects of the bite of a snake vary according to the intensity of the poison.

When the poison is very active, the local irritation is so sudden and so violent, and its effects on the general system are so great, that death soon takes place. When the body is afterwards inspected, the only alteration of structure met with, is in the parts close to the bite, where the cellular membrane is completely destroyed, and the neighbouring muscles very considerably inflamed.

When the poison is less intense, the shock to the general system does not prove fatal. It brings on a slight degree of delirium, and the pain in the part bitten is very severe: in about half an hour swelling takes place from the effusion of serum in the cellular membrane, which continues to increase with greater or less rapidity for about twelve hours, extending during that period into the neighbourhood of the bite; the blood ceases to flow in the smaller vessels of the swoln parts; the skin over them becomes quite cold, the action of the heart is so weak, that the pulse is scarcely perceptible, and the stomach is so irritable, that nothing is retained on it. In about 60 hours these symptoms go off, inflammation and suppuration tion take place in the injured parts, and when the abscess formed is very great, it proves fatal. When the bite has been in the finger, that part has immediately mortified. When death has taken place under such circumstances, the absorbent vessels and their glands have undergone no change similar to the effect of morbid poisons, nor has any part lost its natural appearance, except those immediately connected with the abscess.

In those patients who recover with difficulty from the bite, the symptoms produced by it go off more readily, and more completely, than those produced by a morbid poison, which has been received into the system.

The violent effects which the poison produces on the part bitten, and on the general system, and the shortness of their duration, where they do not terminate fatally, has frequently induced the belief, that the recovery depended on the medicines employed: and in the East Indies, eau de luce is considered as a specific for the cure of the bite of the cobra di capello.

There does not appear to be any foundation for such an opinion; for when the poison is so intense, as to give a sufficient shock to the constitution, death immediately takes place, and where the poison produces a local injury of sufficient extent, the patient also dies, while all slighter cases recover.

The effect of the poison on the constitution is so immediate, and the irritability of the stomach is so great, that there is no opportunity of exhibiting medicines till it has fairly taken place, and then there is little chance of beneficial effects being produced.

The only rational local treatment to prevent the secondary mischief, is making ligatures above the tumefied part, to compress the cellular membrane, and set bounds to the swelling, which only spreads in the loose parts under the skin; and scarifying freely the parts already swoln, that the effused serum may escape, and the matter be discharged, as soon as it is formed.—Ligatures are employed in America, but with a different view, namely, to prevent the poison being absorbed into the system.

